# Mental Health Conditions and Suicide Risk Among Costa Rican University Students

**DOI:** 10.15649/cuidarte.3427

**Published:** 2024-05-29

**Authors:** Daniel Martínez-Esquivel, Patsy Quesada-Carballo, Yerlin Quesada-Rodríguez, Ana Laura Solano-López, Derby Muñoz-Rojas

**Affiliations:** 1 Universidad de Costa Rica, San José, Costa Rica. daniel.martinezesquivel@ucr.ac.cr Universidad de Costa Rica Universidad de Costa Rica San José Costa Rica daniel.martinezesquivel@ucr.ac.cr; 2 Complaint investigator. Smith & Nephew. patsypatt96@gmail.com Smith & Nephew patsypatt96@gmail.com; 3 Safety Data Management Specialist. Pfizer. yerlin.qr23@gmail.com Pfizer yerlin.qr23@gmail.com; 4 Universidad de Costa Rica, San José, Costa Rica. analaura.solanolopez@ucr.ac.cr Universidad de Costa Rica Universidad de Costa Rica San José Costa Rica analaura.solanolopez@ucr.ac.cr; 5 Universidad de Costa Rica, San José, Costa Rica. derby.munoz@ucr.ac.cr Universidad de Costa Rica Universidad de Costa Rica San José Costa Rica derby.munoz@ucr.ac.cr

**Keywords:** Anxiety, Social Support, Depression, Students, Suicide, Ansiedad, Apoyo Social, Depresión, Estudiantes, Suicidio, Ansiedade, Apoio Social, Depressão, Estudantes, Suicídio

## Abstract

**Introduction:**

University students are exposed to academic demands that could impact mental health and trigger suicidal behaviors.

**Objective:**

To analyze the mental health conditions (depression, anxiety, and social support) and suicide risk in Costa Rican university students.

**Materials and Methods:**

Correlational, predictive, and cross-sectional research was conducted. A census sample of 76 university students was included. Variables encompassed sociodemographic data, social support measured by the Multidimensional Scale of Perceived Social Support, depression measured by the Beck Depression Inventory-II, anxiety measured by the Beck Anxiety Inventory, and suicide risk measured by the Plutchik Suicide Risk Scale. An online survey was used. Descriptive analysis, variance analysis, Pearson correlation, and multiple linear regression were performed. Significance level set at p<0.05.

**Results:**

The student body had a mean age of 19.43 years ±1.75, with gender identity evenly distributed. 38.20% reported moderate-severe symptoms of depression, and 32.90% indicated suicide risk. Differences were observed between gender identity in terms of social support and anxiety (p<0.05). Correlations were found between mental health conditions and suicide risk (p=0.001). Depression was identified as a factor contributing to an increased risk of suicide (p=0.001).

**Discussion:**

Previous literature confirms that mental health conditions such as low social support, depression, and anxiety in university students would predispose to the suicide risk.

**Conclusions:**

There is a segment of the university student population experiencing adverse mental health conditions and suicide risk, for whom care strategies focused on their needs should be developed.

## Resumen

**Introducción:**

El estudiantado universitario está expuesto a demandas académicas que podrían afectar la salud mental e incitar las conductas suicidas.

**Objetivo:**

Analizar las condiciones de salud mental (depresión, ansiedad y apoyo social) y el riesgo de suicidio en estudiantes universitarios costarricenses.

**Materiales y Métodos:**

Investigación correlacional, predictiva y transversal. Muestra censal de 76 estudiantes universitarios. Las variables fueron datos sociodemográficos, apoyo social medido por la Escala Multidimensional de Apoyo Social Percibido, depresión medida por el Inventario de Depresión de Beck-II, ansiedad medida por el Inventario de Ansiedad de Beck y riesgo de suicidio medido por la Escala de Riesgo Suicida de Plutchick. Se utilizó una encuesta en línea. Se realizó análisis descriptivo, análisis de varianza, correlación de Pearson y regresión lineal múltiple. Nivel de significancia p<0,05.

**Resultados:**

El estudiantado tuvo una edad media de 19,43 años ±1,75, la identidad de género se distribuyó igualitariamente. El 38,20% manifestó síntomas moderado-severo de depresión, 32,90% refirió riesgo de suicidio. Hubo diferencias entre identidad de género según apoyo social y ansiedad (p<0,05). Se presentaron correlaciones entre las condiciones de salud mental y el riesgo de suicidio (p=0,001). La depresión determinó un aumento del riesgo de suicidio (p=0,001).

**Discusión:**

La literatura previa confirma que condiciones de salud mental como bajo apoyo social, depresión y ansiedad en estudiantes universitarios suscitarían el riesgo de suicidio.

**Conclusiones:**

Existe un sector del estudiantado universitario que presenta condiciones de salud mental adversas y riesgo de suicidio, para el que se debe desarrollar estrategias de cuidado centradas en sus necesidades.

## Resumo

**Introdução:**

Estudantes universitários estão expostos a demandas acadêmicas que podem afetar a saúde mental e incitar ao comportamento suicida.

**Objetivo:**

Analisar as condições de saúde mental (depressão, ansiedade e apoio social) e o risco de suicídio em estudantes universitários da Costa Rica.

**Materiais e Métodos:**

Pesquisa correlacional, preditiva e transversal. Amostra censitária de 76 estudantes universitários. As variáveis foram dados sociodemográficos, apoio social medido pela Escala Multidimensional de Suporte Social Percebido, depressão medida pelo Inventário de Depressão de Beck-II, ansiedade medida pelo Inventário de Ansiedade de Beck e risco de suicídio medido pela Escala de Risco de Suicídio de Plutchick. Foi utilizada uma pesquisa online. Foram realizadas análise descritiva, análise de variância, correlação de Pearson e regressão linear múltipla. Nível de significância p<0,05.

**Resultados:**

O corpo discente tinha idade média de 19,43 anos ±1,75, a identidade de gênero estava distribuída igualmente. 38,20% apresentaram sintomas de depressão moderados a graves, 32,90% relataram risco de suicídio. Houve diferenças entre identidade de gênero segundo suporte social e ansiedade (p<0,05). Foram apresentadas correlações entre condições de saúde mental e risco de suicídio (p=0,001). A depressão determinou risco aumentado de suicídio (p=0,001).

**Discussão:**

A literatura anterior confirma que condições de saúde mental como baixo apoio social, depressão e ansiedade em estudantes universitários aumentariam o risco de suicídio.

**Conclusões:**

Existe um setor de estudantes universitários que apresenta condições adversas de saúde mental e risco de suicídio, para o qual devem ser desenvolvidas estratégias de cuidado focadas em suas necessidades.

## Introduction

Suicide is the second leading cause of death among young people worldwide^1^. It is a complex phenomenon that is part of a continuum of responses known as suicidal behaviors^1^. Suicidal behaviors encompass a range of maladaptive actions divided into four categories: 1) suicidal ideation refers to (self-)reported thoughts about death; 2) suicide threats are warnings that a person plans to take their own life; 3) suicide attempts involve self-directed actions that lead to death; and 4) suicide is the intentional act of ending one's own life^1^.

It is recognized that suicidal behaviors are influenced by multiple factors related to health, society, and the environment, which could affect anyone indiscriminately but are more prevalent in individuals aged 15 to 29, the age range where the university population is concentrated^2^. University students experience a life stage filled with challenging transformations at the personal, interpersonal, and academic levels. They confront a demanding environment that requires responsibility in fulfilling obligations throughout the course of their academic program. At times, these obligations could become a stressful element that, coupled with the tension of daily life, can affect their overall health and lead to the development of a perception of low social support, depressive symptoms, anxiety, and suicidal behaviors^3^.

Regarding social support, a study with university students reports that when individuals do not receive security from family, friend(s), and the community, it is associated with the risk of suicide^4^. Situations such as having a poor relationship with classmates, lacking a functional family relationship, or being concerned about economic status can lead to feelings of loneliness, inexpressiveness, or unfavorable attitudes towards seeking help^4^.

In terms of depression, previous literature has demonstrated the association between suicidal behaviors in university students. It has been noted that the presence of depressive symptoms, such as feelings of sadness or disinterest in enjoyable activities that diminish functional ability, increases the likelihood of suicidal ideation 2.6 times^5^. Regarding anxiety, understood as an autonomous emotional response to a diffuse threat^6^, research on risk factors for the occurrence of suicidal ideation and suicide attempts reported that when anxiety interferes with daily activities, it becomes a predictor of suicidal behaviors in young people^6^.

Associations have been established between depression, anxiety, and poor relationships with loved ones in university students, influencing their coping strategies in maladaptive ways^7^. Significant negative relationships have also been demonstrated between social support and depression, with social support moderating the effect of stressors on students. Social support thus becomes a protective factor for mental health that could have an impact on a better transition to this stage and consequently reduce the aforementioned mental health conditions: depression, anxiety, and suicide risk^8^.

Furthermore, when examining attitudes based on gender identity, they could significantly increase susceptibility to self-harming acts with theintention ofdying(suicide risk),as well asadversemental health conditions such as low social support, depression, or anxiety^2^. In this regard, traditional expectations of masculinity would be linked to significant factors for suicide risk in men, such as depressive symptoms and impulsiveness^9^. Subsequently, expected feminine roles mediate significant factors for suicidal behaviors in women, including depressive symptoms, self-harm, and body dissatisfaction^9^.

In Costa Rica, risk factors for suicidal behaviors in nursing students have been examined, focusing on mental health conditions such as depression, alcohol consumption, stress, and social support^10^. However, suicide risk has not been measured, and associations with mental health conditions have not been explored in university students across various courses. Analyzing these relationships is crucial for a deeper understanding of the issue within the university context and for the development of differentiated, evidence-based strategies for promoting mental health and preventing suicidal behaviors, thus grounded in Mental Health Nursing (MHN) practices.

For these reasons, the objective is to analyze the mental health conditions (depression, anxiety, and social support) and suicide risk in Costa Rican university students. The following hypotheses are proposed:

- H1 There are differences by gender identity in mental health conditions and suicide risk among university students.
- H2 There are relationships between mental health conditions and suicide risk in university students.
- H3 Mental health conditions determine an increase in suicide risk in university students.

## Materials and Methods

The researchers adhered to the guidelines for reporting observational studies in the Strengthening the Reporting of Observational Studies in Epidemiology (STROBE) guide^11^.

A correlational, predictive, and cross-sectional study was conducted at a regional branch of a public higher education institution located in a coastal area ofCosta Rica. Data collection concluded in the year 2022. The population consisted of students from the Integrated General Studies System (SEG), which provides humanistic education and general knowledge to students from various disciplines, usually in the early years of their program. At the time of this study, SEG had approximately 320 enrolled students.

Inclusion criteria were defined as being between 18 and 24 years old and enrolled in at least one SEG course. Exclusion criteria included being enrolled simultaneously at the regional branch and another location or being enrolled in another university (public or private). Data from 6 individuals who, despite meeting eligibility criteria, did not complete the survey in its entirety were excluded.

The sample was census-based, and participant recruitment remained active within designated time frames until achieving adequate statistical power. With the participation of 76 university students, a post hoc analysis using the G.Power 3.1.9.2 program identified a statistical power of 0.98. Once it was confirmed that the study had reached the required number of participants for statistically significant inferences (p<0.05), recruitment was halted.

Study variables included sociodemographic characteristics, social support, depression, anxiety, and suicide risk. Data were collected using a self-administered online survey created on the LimeSurvey platform, a professional tool for online data collection. It was divided into five parts:

1. Sociodemographic Characteristics: Identified age, gender identity, province of residence, and marital status.
2. Multidimensional Scale of Perceived Social Support^12^: Measured an individual's perception of social support in three areas - family, friends, and partner. Comprising 12 questions, with 4 items per area, such as "My friends really try to help me," "I can talk about my problems with my family," or "There is a special person in my life who cares about my feelings." It uses a Likert scale with 7 possible responses, scored from 1 (very strongly disagree) to 7 (very strongly agree). The total score ranges from 12 to 84, with higher scores indicating better perceived social support. Each area is scored from 4 to 28, with higher scores suggesting a better perception of support from family, friends, or a partner. This instrument reported a reliability of a=0.880^12^ previously, and for this study, it demonstrated a Cronbach's Alpha of 0.990.
3. Beck Depression Inventory-II^13^: A scale for measuring depressive symptoms over the past 2 weeks. Consisting of 21 sets of questions evaluating symptoms like "Sadness," "Crying," or "Irritability." It offers 4 response options with variable scores based on symptom intensity, ranging from 0 (absence or mild symptom) to 3 (dysfunctional presence of the symptom). The total sum of responses suggests the level of depression. For Costa Rica, there are proposed cutoff points to establish ranges: minimal from 0 to 7; mild from 8 to 20; moderate from 21 to 33; and severe from 34 to 63^14^. Adapted and validated for Costa Rica with a reliability of a=0.908^14^, this study reported a=0.936.
4. Beck Anxiety Inventory^15^: An instrument that assesses the presence of anxiety symptoms in the last week. Organized into 21 items presenting anxiety symptoms such as "Heated," "Unstable," or "Nervous," with 4 response options ranging from No (0) to Severe (3). The total score is the sum of all responses, with a minimum of 0 and a maximum of 63. This inventory has a cutoff point of 26 indicating severe anxiety. A higher score indicates more significant anxiety symptoms according to proposed levels: minimal from 0 to 7, mild from 8 to 15, moderate from 16 to 25, and severe from 26 to 63. It previously reported an internal consistency of a=0.920^15^, and in this study, a Cronbach's Alpha of 0.905 was obtained.
5. Plutchik Suicide Risk Scale^16^: Evaluates the risk of suicide, allowing differentiation of individuals with suicide risk. Consisting of 15 closed-ended questions such as 'Do you have little interest in socializing with people?', 'Do you see your future as hopeless?' or 'Have you ever tried to take your own life?' The response options are 0 (no) and 1 (yes), with a minimum score of 0 and a maximum of 15. Higher scores indicate greater risk factors. A cutoff point of 6 is suggested to determine suicide risk^17^. In this analysis, a distinction was made between low suicide risk from 0 to 6 and high suicide risk from 7 to 15. The scale previously reported a reliability of a=0.840^16^. In this study, a Kuder-Richardson coefficient of 0.816 is reported.

For participant recruitment, an invitation message along with the online survey link and an infographic providing information about the project was shared with the SEG coordinator. The coordinator disseminated this information via the virtual environment of each SEG course and/or the institutional email of each student. At no point did we have access to sensitive information from individuals interested in collaborating. The obtained information was downloaded and organized into a database in Microsoft Excel 16.69.1. This database was then exported to the Statistical Package for the Social Sciences version 29.0.1.0 (IBM SPSS 29) for statistical analysis by two researchers to enhance precision. The database was stored in Mendeley Data^18^.

Frequencies, measures of central tendency, and measures of variability were estimated to characterize the sample. Analysis of variance (ANOVA) was used to establish differences by gender identity, and Pearson's coefficient was used to calculate associations between variables. In summary, a model was proposed in which suicide risk (dependent variable) was influenced by depression, anxiety, and social support (independent variables). According to the theory, gender identity was selected as an adjusting factor, given its close association with suicidal behaviors and its potential role as a confounding variable. The model was tested using hierarchical multiple linear regression analysis. No assumption violation for this analysis was confirmed. A cut-off for the significance level of p<0.05 was defined. The internal consistency of the scales was analyzed using Cronbach's Alpha coefficient and the Kuder-Richardson coefficient. No missing values were reported.

The research team adhered to ethical principles and good practices to ensure respect for human rights. University students were provided with informed consent to either accept or decline participation in the research. The protocol was approved with code 840-C0-338 by the Scientific Ethics Committee of the University of Costa Rica.

## Results

The participating university students (76) had an average age of 19.43 years ±1.75. Interestingly, gender identity was evenly distributed between male and female, with no one identifying as transgender or other. The majority, 38.20% (29), resided in Puntarenas (the location of the university campus), and 94.70% (72) were single.

Concerning the examined variables, it was observed that 17.20% (13) very strongly disagreed, strongly disagreed, or disagreed regarding perceived social support. Additionally, 38.20% (29) exhibited symptoms of moderate to severe depression. Furthermore, 19.70% (15) reported severe anxiety symptoms, and 32.90% (25) indicated suicide risk. The detailed distributions of sociodemographic data, mental health conditions, and suicide risk are presented in Table 1.

**Table 1:** Distribution of Sociodemographic Data, Mental Health Conditions, and Suicide Risk among the Participants from the Integrated General Studies System (n=76)

Variable	Total		High suicide risk		Low suicide risk		p-value
	n(76)	%	n(25)	%	n(51)	%	
Age							0.968
18-20	61	80.30	20	80.00	41	80.40	
21-24	15	19.70	5	20.00	10	19.60	
Gender identity							0.464
Male	38	50.00	14	56.00	24	47.10	
Female	38	50.00	11	44.00	27	52.90	
Province of domicile							0.938
San José	25	32.90	9	36.00	16	31.40	
Alajuela	2	2.60	1	4.00	1	2.00	
Cartago	4	5.30	1	4.00	3	5.90	
Heredia	2	2.60	1	4.00	1	2.00	
Guanacaste	1	1.30	0	0.00	1	2.00	
Puntarenas	29	38.20	8	32.00	21	41.20	
Limón	13	17.10	5	20.00	8	15.70	
Marital status							0.454
Single	72	94.70	23	92.00	49	96.10	
Free Union	4	5.30	2	8.00	2	3.90	
Social Support							0.001
Very strongly disagree	6	7.90	5	20.00	1	2.00	
Strongly disagree	5	6.60	3	12.00	2	3.90	
Disagree	2	2.70	2	8.00	0	0.00	
Neither agree nor disagree	9	11.80	2	8.00	7	13.70	
Agree	15	19.70	8	32.00	7	13.70	
Strongly agree	14	18.40	3	12.00	11	21.60	
Very strongly agree	25	32.90	2	8.00	23	45.10	
Depression							0.001
Minimal	17	22.40	0	0.00	17	33.30	
Mild	30	39.50	4	16.00	26	51.00	
Moderate	16	21.10	8	32.00	8	15.70	
Severe	13	17.10	13	52.00	0	0.00	
Anxiety							0.001
Minimal	20	26.30	1	4.00	19	37.30	
Mild	21	27.60	5	20.00	16	31.40	
Moderate	20	26.40	9	36.00	11	21.60	
Severe	15	19.70	10	40.00	5	9.80	

Note: n: absolute frequencies; %: relative frequencies; p-value Pearson Chi-Square and Fisher's Exact Test

In a comparative analysis between gender identity related to mental health conditions and suicide risk, significant differences were reported concerning social support and anxiety (Table 2). Male participants exhibited higher social support and more anxiety than female participants.

**Table 2:** Comparison According to Mental Health Conditions and Suicide Risk among the Gender Identity of the Participants from the Integrated General Studies System (n=76)

Variable	Total	Male (n=38)		Female (n=38)		p-value
	X±DE	X±DE	(IC 95%)	X±DE	(IC 95%)	
Social support	61.75 ±22.66	66.95 ±20.24	(60.29-73.60)	56.55 ±24.00	(48.66-64.44)	0.045
Depression	18.67 ±13.03	19.71 ±14.43	(14.97-24.45)	17.63 ±11.58	(13.83-21.44)	0.491
Anxiety	15.88 ±11.43	18.89 ±12.81	(14.68-23.11)	12.87 ±9.08	(9.88-15.85)	0.021
Suicide risk	5.11 ±2.92	5.11 ±3.29	(4.03-6.19)	5.11 ±2.57	(4.26-5.95)	1.000

Note: X=mean; SD=standard deviation; CI=confidence interval; p-value ANOVA

Additionally, Table 3 shows significant correlations between the study variables. Social support shows a moderate significant negative correlation with depression and suicide risk, that is, the greater the social support, the fewer depressive symptoms and suicide risk. Depression shows a strong positive significant correlation with anxiety and suicide risk, that is, the greater the presence of depressive symptoms, the greater anxiety and suicide risk. For its part, anxiety has a strong significant positive correlation with the risk of suicide, that is, the greater the anxiety, the greater the risk of suicide.

**Table 3:** Correlation of Mental Health Conditions and the Risk of Suicide in the Participating Students of the Integrated General Studies System (n=76)

Variable	1	2	3	4
1. Social support	1			
2. Depression	-0.465^*^	1		
3. Anxiety	-0.088	0.641^*^	1	
4. Suicide risk	-0.450^*^	0.772^*^	0.590^*^	1

Note: ^*^p<0.01 (two-tailed) Pearson coefficient

Finally, the effect of mental health conditions on suicide risk was examined (Table 4). Depression had a significant effect on suicide risk, indicating that an increase in depression symptoms intensifies the suicide risk (B=0.623; p=0.001). In contrast, anxiety and social support showed no effect on suicide risk.

**Table 4:** Regression Model for the Effect of Mental Health Conditions on Suicide Risk in the Participants from the Integrated General Studies System (n=76)

Variable	F	R2	ß	(IC 95%)	p-value
Depression	37.129	0.677	0.623	(0.09-0.19)	0.001
Anxiety			0.191	(-0.01-0.10)	0.056
Social support			-0.124	(-0.04-0.01)	0.137
Gender identity (Male)			0.072	(-0.43-1.26)	0.328

Note: ß=standardized beta coefficient

## Discussion

The results obtained could provide answers to the objective and the hypotheses raised. It is observed that there is a percentage of the group of participants where there are alterations in mental health conditions such as low social support, moderate-severe symptoms of depression, severe symptoms of anxiety and high risk of suicide that endanger their health, including their life. When making comparisons by gender identity, significant differences are observed according to social support and anxiety, where male participants presented higher scores. Significant positive correlations are exhibited between depression, anxiety and suicide risk, and inverse correlations with social support. In addition, greater symptoms of depression determined an increased risk of suicide. Therefore, H2 is fully accepted and H1 and H3 are partially accepted.

The years at the university are a challenge that would offer positive aspects that reward personal and professional development, and the student body’s self-realization. However, for many young people they tend to be very threatening and stressful years. When adequate coping resources are not available, these experiences would affect health, inciting the appearance of physical and mental illnesses, or maladaptive strategies such as suicidal behaviors^19^.

Consequently, it has been argued that university students could have a greater risk of suicide during their study stay due to circumstances linked to this fact such as family separation, financial problems or complicated relationships. Collectively, such difficulties would give rise to adverse mental health conditions such as low social support, depression, and anxiety, which justifies what the participants in this study expressed^20^.

The results show that there is a sector of the sample that would experience a negative perception of the support they receive, along with more depression and anxiety symptoms, as well as higher suicide risk factors. Findings align with other research that identifies family problems and relationships that generate insecurity, hopelessness, depression, anxiety, and academic stress as major components of suicidality^21^.

Therefore, when individuals do not feel connected to others or lack a sense of belonging in a given environment, they may become frustrated. Frustration may indicate a failure in interpersonal relationships, leading to a burden of social isolation due to disconnection between individuals.

Moments without significant connections could affect mood, influencing depression and impacting suicidal behaviors^22^.

Furthermore, it has been discussed that suicidal behaviors are linked to established gender norms in society. In this regard, significant differences are noted between gender identity regarding social support and anxiety, suggesting a better perception of social support and higher anxiety symptoms among men, in line with another analysis^23^. In contrast, despite the expectation that women would have a better support network or face more negative life situations, the results may be related to potential changes in current masculine traits^23^.

It is noteworthy that no differences were found regarding depression and suicide risk between gender identities, which is contrary to other research indicating that the risk of depression in women tends to be amplified in the current context, leading to comorbidity with suicide risk^24^. It is emphasized that society is constantly evolving, and for this topic, it is crucial to study each community without prejudice, openly, to become aware of their needs and their members’ responses.

The detected findings allowed inferring significant correlations between depression, anxiety, and suicide risk, which is reasonable considering that both depression and anxiety have been identified as the most important predictors for suicidal behaviors^25^. Consequently, there is similarity with another analysis demonstrating significant associations between these variables for university students, where 18.20% and 13.60% of individuals with a suicidal history had symptoms of depression and anxiety, respectively^25^.

It is considered that these conditions are delicate for the mental health of this population, and careful examination is needed to determine if they are determinants for suicidal acts, as the phenomenon should be investigated comprehensively to achieve a better understanding. With this, the intention is to depathologize intervention proposals for more holistic approaches in line with human values.

A similar situation occurs with social support, where its inverse association has agreed with the results of another study showing a negative relationship with depression. Therefore, social support could be one of the compelling reasons to abandon the idea of suicide as a way to solve a problem^25^^,^^26^. It is considered a protective factor for individuals experiencing distress who need to communicate it to others. For this reason, it would be appropriate to promote its strengthening through various actions from the university and the community, reducing social stigma around suicidal behaviors and fostering a positive sense of identity^27^.

In conclusion, the data demonstrated an effect of depression on the increased suicide risk. This aligns with previous literature establishing that one of the strongest predictors of suicide is adjacent mental health problems such as depression or anxiety. It is estimated that in Europe and North America, 98.00% of suicide cases were diagnosed with psychiatric disease. Moreover, published results indicate that around 21.40% of students who die by suicide have mood disorders such as depression. Likewise, it is known that symptoms of lack of support and depression have strong effects on the increased suicide rate in students^28^^,^^29^.

The findings from this research are useful for Mental Health nurses and related professionals as they contribute to the mental health agenda, strengthening suicide prevention. Proper use of these findings could enhance the ability to address the needs of youth in this area through evidence-based therapeutic activities^30^.

### Strengths

It contributes to MHN and other disciplines interested in caring for individuals by approaching the reality of the mental health experiences of university students immersed in a specific context, providing insight into the current state of mental health in this group. The interpretation carried out suggest relevant information that could guide intervention strategies for addressing suicidal behaviors in the participants.

### Limitations

As limitations, it is emphasized that the results should not be generalized due to the sample size and data collection being restricted to a single regional campus and a single university. The recruitment strategy might be vulnerable to self-selection biases as there is a possibility that individuals with certain characteristics were more willing to participate than others. Additionally, the online nature of the survey could potentially affect the precision of measurements if there was a tendency to respond in a similar way to questions considered intimate. The results should be interpreted with caution as there could be spurious associations between variables due to unmeasured confounding biases.

### Recommendations

It is advised for future research to develop longitudinal studies that allow for understanding changes and transitions in suicidal behaviors, as well as e-health investigations for the development of counseling technologies tailored to the preferences of young people. There is a need to decentralize the observation of this phenomenon to understand the people’s stories in different locations or regions with distinct opportunities. Interdisciplinary professionals should participate in discussions on the topic.

Recommendations for the practice of MHN encourage consideration of the results presented in this study to formulate policies, plans, and programs for the support of students in the academic environment of the regional headquarters, prioritizing the development of promotion strategies that involve family members, friends, and partners in raising awareness of comprehensive mental health. It is also important that care should be focused on the real needs of the person to ensure its effectiveness and efficiency. Health care personnel training related to addressing mental health issues is also a necessity.

## Conclusion

The study concludes that suicidal behaviors are a maladaptive human response not uncommon among university students. In this specific population, there is a group exhibiting factors such as depression and anxiety that are linked to the risk of suicide, and this risk does not vary significantly by gender identity. Conversely, social support appears to act as a protective element, counteracting the negative impact of adverse mental health conditions and reducing suffering. Finally, it is emphasized that increased symptoms of depression are associated with a higher suicide risk.
